# The Covering of Exposed Pulps

**Published:** 1881-01

**Authors:** H. Thompson


### THE DENTAL REGISTER.

VOL. XXXV.] JANUARY, 1881. [No. 1.

### THE COVERING OF EXPOSED PULPS.

*Translated from A. Witzel on Pulp Treatment, by H. Thompson.*

FOR a long time earnest attempts have been made in conservative dentistry not, as formerly, to destroy* the exposed and seemingly healthy pulp by escharotics; but, if at all possible, to preserve it for the tooth. The method, namely to again protect a pulp, which has been accidentally exposed, by a cap, was recommended twenty years ago by several able colleagues, and practically demonstrated. For this purpose there were used, as we have already remarked — as long as the causes of the inflammation of the pulp and the conditions under which these organs could again become healthy were not known — the most varied materials to protect the exposed pulp against the "Pressure of the filling," to which had always been ascribed the bad result of this operation.

A more rational procedure for the purpose of covering the pulp was recommended fifteen years ago by Fricke,† (from Lueneburg). He covered the pulp, after treating it with *creosote*, with the well known chloride of zinc cement. This method, which rests upon the only correct principle, namely: to give to the exposed pulp a closely fitting protective covering, was, as so many other things first taken notice of by the German colleagues, when it was imported into Germany as the "Great American

* By exposed pulps we understand those which we have uncovered with our instruments, accidentally or intentionally.

† At the meeting of the Central Society of German Dentists at Frankfort on Main, in the year 1863.

invention," the so called "Atkinson's Method." Since that time the covering of the exposed pulp, by means of chloride of zinc cement, occupies a prominent position in Conservative Dentistry, and it is not to be denied that certain successful issues have been gained by it. This method depends, as we have already mentioned, upon this, that a closely fitting protective covering is to be brought to the exposed pulp without any intervening space; and this is indeed the cardinal rule concerning the operation under discussion.

Unfortunately, the method of Fricke is only then applicable, at least, can only be carried through with tolerable result, when the exposed pulp is quite healthy, and the small exposed portion has not been injured. In all other cases, and these are by far more numerous, more pulps have been destroyed by the chloride of zinc coverings than preserved. That the pulp does not give pain for a time is not reliable evidence that the preservation of this organ will be secured; but that the pulp should functionate normally under the cement covering. This favorable result is hardly obtained in forty per cent. In all the rest of the cases the wounded pulp crowns are destroyed by shrinking under the covering of chloride of zinc cement; a circumstance which I, together with Tomes ascribe, next to the stopping of the pulp by means of chloride of zinc, to the hygroscopic quality of the zinc cement. From the pulp there is extracted, during the hardening of the cement, the moisture so forcibly, that necessarily pain and shrinking of the wounded parts must ensue. Further, the Chloride of Zinc is a deep reaching escharotic, the effect of which upon the inflamed pulps we can study very well. Here the remedy, even if we have previously made the painful surface of the pulp insensible by means of *Phenol-tannin* so that this even bears slight pressure with the probe, causes so strong a reaction in the parachyma of the pulp crown, which is rich in nerves and vessels, that the healing of the pulp wound so treated is never to be thought of. The pulp crowns thus saturated with chloride of zinc, shrink in favorable cases under the cover, and thus give the best opportunity for the destruction of the healthy parts, and indirectly for periostitis.

The action of the chloride of zinc is entirely analogous, where it reaches the diseased pulp through a thin or softened layer of dentine, except the attacks of pain come on much more severely in a pulpitis, which, until this time had been latent after the employment of the chloride of zinc, in the closed cavity, than if the organ had been previously exposed, a point to which I wish to direct the attention, especially of those, who perhaps without distinction, fill every tender cavity temporarily with cement.

In the process of *covering* healthy pulps by means of chloride of zinc cement, the pain occurs immediately after the contact of the mass with the pulp surface, lasts four or five minutes, and gradually disappears entirely. The action is different in diseased exposed pulps, where we are compelled first to remove the pain, by means of *creosote* or *phenol*. Here immediately after the *covering* the patient experiences no inconvenience, and only after a lapse of ten or twelve minutes, there sets in a pain, which equals any other in severity, and only by the immediate removal of the *cover* the inflammation of the whole pulp can be obviated.

For the action of the chloride of zinc cement, just discussed, the following cases may act as examples.

The pulp from a lower bicuspid tooth of a girl twenty-three years of age. The patient had only slight pain in the tooth, the pulp of which I exposed by removing the carious masses. After treating the pulp, at first, with phenol, I then covered it with chloride of zinc cement, and — because I did not wish to give a particularly favorable prognosis to the experiment — filled it temporarily with gutta-percha. The following day the patient returned to me with severe pain in the filled tooth, and also because it was already tender on percussion, I extracted it immediately. Splitting the tooth I found the exposed part corroded, white and shrunken; the surrounding parts, which had not been brought in direct contact with the chloride of zinc, showed a discoloration of the tissue, which discoloration — including some fine small branches of vessels — extended to the centre of the pulp crown. Here we see in consequence of the etching, (or corroding,) the branches of the vessels very much enlarged, and filled to excess with blood. These extend, as dark red lines, through the entire root pulp, and send off, at certain places, prolongations, also filled to excess with blood. The middle of the specimen is colored red, by the exuded blood in those places in which the calcium spindles, (*Dentinoide*) lie between the vessels. With few exceptions all sore pulps, which have been directly covered with chloride of zinc cement will show at the exposed portions the shrunken and discolored part, and in those cases in which the pains after the covering with cement, may be accounted for by the irritation of the vascular system, already present ; the pathological changes in the vessels here described, may be proven also to exist in the pulp roots.

*Case Second.* Pulp from a lower very carious wisdom tooth, from which the softened dentine layer was not altogether removed ; and which, after treating with phenol and the mastic covering, I filled on the following day with chloride of zinc cement. The patient experienced, after the treatment of this tooth, which had only pained slightly, no inconvenience during the first days. After a few weeks there appeared, as it seemed to me, with the beginning of menstruation, pretty violent, drawing pains in the tooth ; in consequence extraction was undertaken.

Now, here we observe that the root of the pulp is traversed by vessels greatly enlarged, and surcharged with blood, which pierce in the centre of the crown that part which is inflamed and engaged in calcification, and terminate above this as fine ramifications at the periphery of the pulp. In the region of the calcified portions there exists a quantity of air bubbles, which are enclosed in the parenchyma of the shrunken pulp.

Zsigmondy, already calls our attention to this, namely, that if the pain, after the application of the chloride of zinc cap continues longer than a quarter of an hour, the cap is to be removed again, and *creosote* applied until the cessation of the pain. Zsigmondy, however, mistakes the causes of this, which he wishes to trace to a mechanical irritation, *a pressure*, which the cap exercises upon the exposed portion of the pulp, while in my opinion, the pain is brought on by the deeply spreading corrosive action of the chloride of zinc, and only then fails to appear when the affected pulp crown is totally devitalized by the continuous etching with *creosote* and chloride of zinc.

Dr. Zsigmondy's advice, namely, to remove again the cement cap, if the carefully covered pulp still pains, when a quarter of an hour has elapsed, is good and to be encouraged ; yet, I add this: that one should never, after this operation, attempt the *preservation* of such a pulp-crown.

The surface of the pulp, which in a healthy condition is distinguished by a certain elasticity and firmness, so that it is a little sensitive to slight pressure, will — even if the inflammation has existed for a few days — be histologically so changed, that the diseased surface of the pulp is easily traversed and partially destroyed, especially by such corroding substances as chloride of zinc.

It seems suitable to me that I should here insert the microscopic structure of the pulp of the tooth, which I have borrowed from the excellent Pathology of the Teeth, by Wedl. [See Wedl's Pathology of the Teeth, pages 55 and 56. Ed.]

Figure 8. Portion of the border of the tooth pulp from a *colt*, in transverse section (or cut). The dentine cells, arranged side by side, show at their peripheral end freely prominent, thick prolongations torn from the tooth-bone, contain in their finely granular protoplasm one or two oval nuclei, separated more or less, (lessening in diameter like a neck,) and communicate, by means of their pointed central ends with the spindle cells of the parenchyma.

Numerous blood vessels, with their openings, have fallen transversely or obliquely in the section, (four hundred times enlarged).

Figure 9. Transverse section through the pulp of a canine tooth. Let there be observed in this segment the line of dentine cells, with free prominent processes, *a*, numerous bundles of new fibres cut transversely *b*, and capillaries *c*. The vessel openings increase in circumference towards the central portion of the pulp (*d*). A texture of bundles of connective tissue and spindle cells forms the stroma, (enlarged one hundred times). This delicate structure does not endure a forcibly acting corroding substance. If we wish to *cover* with success the exposed pulp, other measures are to be employed than those followed until now, by many practitioners, for attaining to this object, which to *conservative dentistry* must always be a task of the greatest importance.

We must finally cease the indiscriminate treatment of exposed pulps by means of strong corroding substances, by which always a portion of the surface of the pulp is destroyed, we must lastly proceed in a conservative manner.

The employment of weak phenal solutions may and can have here only one object — to destroy the offensive substances on the exposed pulp, that matter which excites decomposition, eventually to remove the hyperæthesia of the odontoblast layers.

A different action we do not require of the substances in question. If the action shows itself otherwise, we destroy, by our rude treatment that layer of the surface of the pulp the preservation of which, before all others, should be our object. I mean the odontoblasts and the underlying spindle cells from which, principally, the repair of the defect in the pulp cavity, by means of supply, dentine is to be expected.

In the covering of exposed pulps, consequently provision is to be made, that: First, the exposed part is not brought into contact with the decomposing products. Before all other things, the slimy secretion, which usually exists at the margin of the gums, must, even before the treatment, be removed with a soft brush, and the gums themselves be painted with a little phenol solution. We must, besides, constantly keep the instruments, with which we usually clean the carious cavity, clean; and moisten them now and then with phenol.

*Second.* For the *covering* only substances should be chosen by means of which the former state, before the exposure, can be possibly restored. The exposed portion is neither allowed to be corroded by the *cap*, nor strongly irritated.

*Third.* The covering must be brought into close apposition with the exposed portion of the pulp; it must also be so strong that it cannot be broken by the pressure which is necessary for the condensation of an *amalgam filling.**

* Teeth, the pulps of which have been exposed, and covered, should never be filled at once with *gold*. See unfavorable results on page 31.

For this purpose there were formerly used small lead, tin or gold leaves, which were attached over the pulp defect by means of gum, and they served as a substratum for the filling. For the last ten years, however, the method of Fricke, mentioned at the beginning, modified more or less, has been employed for covering the pulps.

Muehlreiter treats exposed healthy pulps with a weak carbol solution, and covers the exposed part with a small piece of paper, which is intended to protect the pulp against the corroding action of the subsequent covering of chloride of zinc cement.

W. Suersen employs for the protection of the exposed pulp, a small piece of paper, which he moistens with a solution of chloride of zinc, but which he again dries and covers, likewise, with cement. Zsigmondy and Alexovits advise to paint the exposed portion at first with Traumaticin, and then to *cover* with some chloride of zinc cement.

Again others, especially von Landsdorf, who frequently has already called attention to the detrimental influence of the chloride of zinc *cap*, uses for the purpose of *covering* of healthy pulps some *asbestos* or gutta-percha, sometimes also dry zinc-oxid, and *creosote.*

Certainly, good results can be obtained by the methods named, on condition that the healthy pulp is treated primarily in a judicious manner, and that care is taken that the *cap* lies in close apposition to the exposed portion.

Generally, however, the exposed pulps are no longer (in a healthy state) healthy. In the part which lies nearest the carious defect there can be at least shown to exist already an *hypercœmia* of the capillary vessels, and then, in most cases, the exposed part is somewhat injured.

We have, therefore, before us a wound, and this must — if the pulp should be preserved for the tooth — caused to cicatrise per primum. To attain this, the following procedure, which I hinted at already three years ago, during a discussion of the same theme at Cassel — I refer to my communications made at that time — seems to me to be the most judicious: (these are they): The covering of the pulps with cement, which I shall presently describe more minutely, seems at the first moment to be more particular than it really is; at all events, it offers the greatest number of chances that the wound under the cap may heal well and quickly.

In this chapter I have named these means for the covering of exposed pulps, which, until the present time, have been used in practice with good results; next I shall discuss "The *Indication* and *Technique* of Pulp covering."

